# Poly[μ-aqua-aqua­(μ-benzene-1,2,4,5-tetra­carboxyl­ato)gadolinate(III)potassium(I)]

**DOI:** 10.1107/S1600536811016254

**Published:** 2011-05-11

**Authors:** Xue Yun Gong, Lei Zhang

**Affiliations:** a Department of Physics and Chemistry, Henan Polytechnic University, Jiaozuo, Henan 454000, People’s Republic of China

## Abstract

In the title compound, [KGd(C_10_H_2_O_8_)(H_2_O)_2_]_*n*_, the Gd^3+^ ion is nine-coordinated by eight O atoms from five individual benzene-1,2,4,5-tetra­carboxyl­ate (btec) ligands and one water mol­ecule, and the K^+^ ion is eight-coordinated by six O atoms from five individual btec ligands and two water mol­ecules. In the crystal, the btec half-mol­ecules are completed by crystallographic inversion symmetry. GdO_9_ and KO_8_ polyhedra are connected, forming layers in the *ab* plane, which are further inter­connected by μ_8_- or μ_12_-bridging btec ligands, forming a three-dimensional structure.

## Related literature

For structures based on H_4_btec ligand, see: Huang *et al.* (2009 ▶); Lu *et al.* (2005 ▶); Wu *et al.* (2001 ▶); Zhang *et al.* (2005 ▶). For the isotypic neodymium(III) compound, see: Dai *et al.* (2008 ▶). 
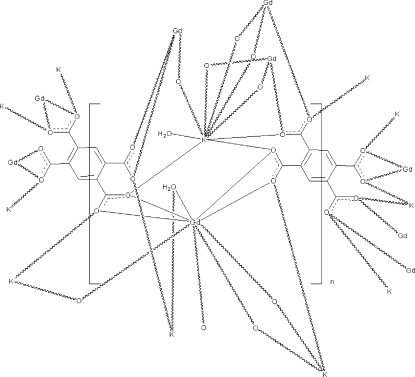


## Experimental

### 

#### Crystal data

[KGd(C_10_H_2_O_8_)(H_2_O)_2_]
*M* *_r_* = 482.50
Monoclinic, 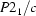

*a* = 8.9003 (1) Å
*b* = 7.7816 (1) Å
*c* = 17.5150 (3) Å
β = 91.857 (1)°
*V* = 1212.43 (3) Å^3^
*Z* = 4
Mo *K*α radiation
μ = 5.87 mm^−1^
*T* = 296 K
0.20 × 0.10 × 0.10 mm

#### Data collection

Bruker SMART APEXII CCD area-detector diffractometer
Absorption correction: multi-scan (*SADABS*; Sheldrick, 1996 ▶) *T*_min_ = 0.386, *T*_max_ = 0.591
14043 measured reflections
3002 independent reflections
2588 reflections with *I* > 2σ(*I*)
*R*_int_ = 0.037

#### Refinement

*R*[*F* ^2^ > 2σ(*F* ^2^)] = 0.024
*wR*(*F* ^2^) = 0.051
*S* = 1.04
3002 reflections
199 parameters
H-atom parameters constrained
Δρ_max_ = 0.91 e Å^−3^
Δρ_min_ = −1.29 e Å^−3^

### 

Data collection: *APEX2* (Bruker, 2008 ▶); cell refinement: *SAINT* (Bruker, 2008 ▶); data reduction: *SAINT*; program(s) used to solve structure: *SHELXS97* (Sheldrick, 2008 ▶); program(s) used to refine structure: *SHELXL97* (Sheldrick, 2008 ▶); molecular graphics: *SHELXTL* (Sheldrick, 2008 ▶); software used to prepare material for publication: *SHELXL97*.

## Supplementary Material

Crystal structure: contains datablocks I, global. DOI: 10.1107/S1600536811016254/si2351sup1.cif

Structure factors: contains datablocks I. DOI: 10.1107/S1600536811016254/si2351Isup2.hkl

Additional supplementary materials:  crystallographic information; 3D view; checkCIF report

## Figures and Tables

**Table 1:** Selected bond lengths (Å)

Gd1—O6^i^	2.337 (3)
Gd1—O7^ii^	2.376 (2)
Gd1—O8	2.433 (2)
Gd1—O3	2.441 (2)
Gd1—O1	2.447 (3)
Gd1—O5^iii^	2.451 (2)
Gd1—O4^iii^	2.503 (2)
Gd1—O9	2.520 (3)
Gd1—O2	2.604 (2)
K1—O2	2.714 (3)
K1—O6	2.783 (3)
K1—O8	2.795 (3)
K1—O10	2.842 (3)
K1—O1^iv^	2.860 (3)
K1—O4^iv^	2.875 (3)
K1—O7^i^	2.891 (3)
K1—O9^v^	2.893 (3)

Symmetry codes: (i) 
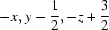
; (ii) 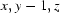; (iii) 
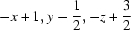
; (iv) 
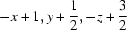
; (v) 
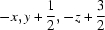
.

**Fig. 1.**
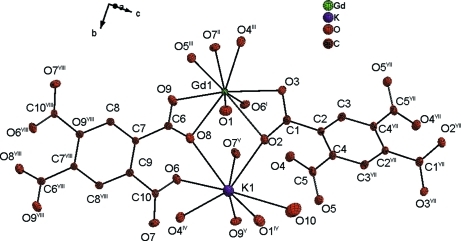
The GdIII and KI coordination environment of the title compound indicating the inversion symmetry of the btec ligands by symmetry codes vii and viii. Displacement ellipsoids are drawn at the 50% probability level for non-H atoms. [Symmetry codes: (i) -x, y - 1/2, -z + 3/2; (ii) x, y - 1, z; (iii) -x + 1, y - 1/2, -z + 3/2; (iv) -x + 1, y + 1/2, -z + 3/2; (v) -x, y + 1/2, -z + 3/2; (vii) -x + 1, -y + 1, -z + 2; (viii) -x, -y + 2, -z + 1].

**Fig. 2.**
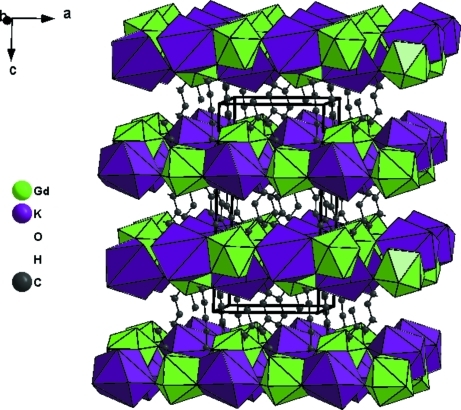
View of the three-dimensional network for the title compound.

## supplementary crystallographic information

### Comment

The btec ligand is a remarkably versatile building block for the construction of supramolecular architectures due to its four rigid carboxyl groups and various coordination modes in the self-assembly reaction (Huang *et al.*, 2009; Lu *et al.*, 2005; Wu *et al.*, 2001; Zhang *et al.*, 2005). Furthermore, they can provide directional conformation of network structures *via* noncovalent contacts like hydrogen bonding and aromatic stacking. In this paper, we report the preparation and crystal structure of a new potassium(I)-gadolinium(III) complex, [KGd(btec)(H_2_O)_2_]_n_, where btec = benzene-1,2,4,5-tetracarboxylate. The crystal structures of a Nd^iii^analogue has been reported recently (Dai *et al.*, 2008).

As shown in Fig. 1, the asymmetric building unit of title compound comprises one Gd^III^ atom, one K^I^ atom, two coordinated water molecules and two btec ligands. The two crystallographic distinct btec ligands occupy inversion symmetry in the structure. One of them acts as a µ_12_-bridge linking six Gd^III^ and six K^I^ atoms, while the other one acts as a µ_8_-bridge linking four Gd^III^ and four K^I^ atoms. Considering the linking environment of the gadolinium(III) and potassium(I) atoms, Gd^III^ is nine-coordinated by eight O atoms from five individual btec ligands and one water molecule, while the K^I^ atom is eight-coordinated by six O atoms from five individual btec ligands and two water molecules, as listed in Tab. 1. As shown in Fig. 2, when C and H atoms are omitted, a two-dimensional Gd—K—O framework is emerged in the *ab* plane. Furthermore, these two-dimensional layers are integrated by C atoms of btec ligands into a three-dimensional framework.

### Experimental

A mixture of 1,2,4,5-benzenetetracarboxylic (0.05 g), Gd_2_O_3_ (0.05 g), KOH(0.05 g) and H_2_O (15 ml) was heated at 448 K for 7 d in a sealed 25 ml Teflon-lined stainless steel vessel under autogenous pressure. After cooling to room temperature at a rate of 5 C h^-1^, colorless prismatic crystals were obtained in low yield.

### Refinement

The H atoms of C atoms were positioned geometrically and refined with a riding model, with C—H = 0.93 Å and *U*_iso_(H) = 1.2Ueq(C). The water H atoms were located in difference fourier maps, and then refined with a riding model, with O—H = 0.85 and 0.87 Å, and *U*_iso_(H) = 1.2Ueq(O).

### Crystal data

**Table:**

[KGd(C_10_H_2_O_8_)(H_2_O)_2_]	*F*(000) = 916
*M**_r_* = 482.50	*D*_x_ = 2.643 Mg m^−^^3^
Monoclinic, *P*2_1_/*c*	Mo *K*α radiation, λ = 0.71073 Å
Hall symbol: -P 2ybc	Cell parameters from 2537 reflections
*a* = 8.9003 (1) Å	θ = 2.3–25.1°
*b* = 7.7816 (1) Å	µ = 5.87 mm^−^^1^
*c* = 17.5150 (3) Å	*T* = 296 K
β = 91.857 (1)°	Prism, colourless
*V* = 1212.43 (3) Å^3^	0.20 × 0.10 × 0.10 mm
*Z* = 4	

### Data collection

**Table:**

Bruker SMART APEXII CCD area-detector diffractometer	3002 independent reflections
Radiation source: fine-focus sealed tube	2588 reflections with *I* > 2σ(*I*)
graphite	*R*_int_ = 0.037
Detector resolution: 83.33 pixels mm^-1^	θ_max_ = 28.3°, θ_min_ = 2.3°
ω scans	*h* = −11→11
Absorption correction: multi-scan (*SADABS*; Sheldrick, 1996)	*k* = −10→9
*T*_min_ = 0.386, *T*_max_ = 0.591	*l* = −23→23
14043 measured reflections	

### Refinement

**Table:**

Refinement on *F*^2^	Primary atom site location: structure-invariant direct methods
Least-squares matrix: full	Secondary atom site location: difference Fourier map
*R*[*F*^2^ > 2σ(*F*^2^)] = 0.024	Hydrogen site location: inferred from neighbouring sites
*wR*(*F*^2^) = 0.051	H-atom parameters constrained
*S* = 1.04	*w* = 1/[σ^2^(*F*_o_^2^) + (0.0208*P*)^2^ + 1.8584*P*] where *P* = (*F*_o_^2^ + 2*F*_c_^2^)/3
3002 reflections	(Δ/σ)_max_ = 0.002
199 parameters	Δρ_max_ = 0.91 e Å^−^^3^
0 restraints	Δρ_min_ = −1.29 e Å^−^^3^

### Special details

**Table:**

Geometry. All e.s.d.'s (except the e.s.d. in the dihedral angle between two l.s. planes) are estimated using the full covariance matrix. The cell e.s.d.'s are taken into account individually in the estimation of e.s.d.'s in distances, angles and torsion angles; correlations between e.s.d.'s in cell parameters are only used when they are defined by crystal symmetry. An approximate (isotropic) treatment of cell e.s.d.'s is used for estimating e.s.d.'s involving l.s. planes.
Refinement. Refinement of *F*^2^ against ALL reflections. The weighted *R*-factor *wR* and goodness of fit *S* are based on *F*^2^, conventional *R*-factors *R* are based on *F*, with *F* set to zero for negative *F*^2^. The threshold expression of *F*^2^ > σ(*F*^2^) is used only for calculating *R*-factors(gt) *etc*. and is not relevant to the choice of reflections for refinement. *R*-factors based on *F*^2^ are statistically about twice as large as those based on *F*, and *R*- factors based on ALL data will be even larger.

### Fractional atomic coordinates and isotropic or equivalent isotropic displacement parameters (Å^2^)

**Table:**

	*x*	*y*	*z*	*U*_iso_*/*U*_eq_	
Gd1	0.21753 (2)	0.47536 (2)	0.699264 (9)	0.00997 (6)	
K1	0.22962 (11)	0.97212 (11)	0.78942 (5)	0.02529 (19)	
O1	0.4699 (3)	0.5723 (3)	0.66830 (15)	0.0226 (6)	
H1A	0.5081	0.6065	0.7107	0.027*	
H1B	0.4533	0.6604	0.6406	0.027*	
C1	0.3791 (4)	0.5106 (4)	0.8437 (2)	0.0147 (7)	
O2	0.3319 (3)	0.6478 (3)	0.81457 (14)	0.0183 (6)	
C2	0.4485 (4)	0.5076 (4)	0.92329 (19)	0.0128 (7)	
O3	0.3601 (3)	0.3676 (3)	0.81063 (14)	0.0210 (6)	
C3	0.3828 (4)	0.3974 (4)	0.97488 (18)	0.0142 (7)	
H3	0.3041	0.3270	0.9581	0.017*	
O4	0.6594 (3)	0.6935 (3)	0.82750 (13)	0.0180 (6)	
C4	0.5683 (4)	0.6106 (4)	0.94877 (18)	0.0128 (7)	
O5	0.7106 (3)	0.8636 (3)	0.92519 (13)	0.0173 (6)	
C5	0.6512 (4)	0.7290 (4)	0.89702 (19)	0.0139 (7)	
O6	−0.0404 (3)	1.0020 (3)	0.70586 (14)	0.0163 (5)	
C6	0.0794 (4)	0.7479 (4)	0.61673 (19)	0.0129 (7)	
O7	0.0284 (3)	1.2656 (3)	0.67405 (14)	0.0168 (5)	
C7	0.0324 (4)	0.8804 (4)	0.55820 (18)	0.0105 (7)	
O8	0.1973 (3)	0.7726 (3)	0.65718 (14)	0.0189 (6)	
C8	0.0245 (4)	0.8287 (4)	0.48207 (18)	0.0118 (7)	
H8	0.0403	0.7138	0.4700	0.014*	
O9	0.0079 (3)	0.6102 (3)	0.61982 (15)	0.0236 (6)	
C9	0.0067 (4)	1.0533 (4)	0.57629 (19)	0.0121 (7)	
C10	−0.0015 (4)	1.1124 (4)	0.65777 (18)	0.0107 (7)	
H10A	0.3331	1.0155	0.9851	0.013*	
H10B	0.4337	0.9339	0.9384	0.013*	
O10	0.3392 (4)	0.9561 (4)	0.94345 (18)	0.0398 (8)	

### Atomic displacement parameters (Å^2^)

**Table:**

	*U*^11^	*U*^22^	*U*^33^	*U*^12^	*U*^13^	*U*^23^
Gd1	0.01314 (10)	0.00955 (8)	0.00718 (8)	0.00160 (7)	−0.00007 (6)	−0.00017 (6)
K1	0.0245 (5)	0.0240 (4)	0.0270 (5)	−0.0026 (4)	−0.0061 (4)	0.0027 (3)
O1	0.0190 (15)	0.0213 (13)	0.0278 (15)	−0.0025 (11)	0.0022 (12)	−0.0034 (11)
C1	0.0161 (19)	0.0175 (17)	0.0104 (16)	0.0008 (14)	0.0006 (14)	0.0011 (13)
O2	0.0226 (15)	0.0168 (13)	0.0153 (13)	−0.0012 (11)	−0.0046 (11)	0.0045 (10)
C2	0.0161 (19)	0.0125 (16)	0.0096 (16)	0.0008 (13)	−0.0010 (13)	0.0008 (12)
O3	0.0335 (17)	0.0156 (12)	0.0133 (13)	0.0060 (11)	−0.0071 (12)	−0.0033 (10)
C3	0.0158 (19)	0.0157 (17)	0.0111 (16)	−0.0033 (14)	−0.0003 (14)	0.0000 (13)
O4	0.0225 (15)	0.0206 (13)	0.0109 (12)	−0.0066 (11)	0.0026 (11)	0.0019 (10)
C4	0.0151 (19)	0.0140 (17)	0.0092 (16)	0.0013 (14)	0.0001 (14)	0.0018 (13)
O5	0.0225 (15)	0.0175 (12)	0.0121 (12)	−0.0048 (11)	0.0011 (11)	0.0015 (10)
C5	0.0122 (18)	0.0161 (17)	0.0134 (17)	0.0005 (14)	0.0003 (14)	0.0050 (13)
O6	0.0220 (14)	0.0159 (13)	0.0111 (12)	0.0023 (10)	0.0044 (10)	0.0046 (9)
C6	0.020 (2)	0.0116 (16)	0.0075 (15)	0.0030 (14)	0.0051 (14)	−0.0011 (12)
O7	0.0199 (15)	0.0144 (12)	0.0161 (13)	−0.0036 (10)	0.0023 (11)	−0.0040 (10)
C7	0.0137 (18)	0.0095 (15)	0.0083 (15)	−0.0020 (13)	0.0009 (13)	0.0015 (12)
O8	0.0193 (15)	0.0172 (13)	0.0199 (14)	0.0003 (11)	−0.0055 (11)	0.0045 (10)
C8	0.0151 (19)	0.0109 (16)	0.0096 (16)	0.0013 (13)	0.0026 (13)	−0.0005 (12)
O9	0.0280 (17)	0.0165 (13)	0.0255 (15)	−0.0050 (11)	−0.0090 (12)	0.0082 (11)
C9	0.0133 (18)	0.0117 (16)	0.0112 (16)	−0.0010 (13)	−0.0001 (13)	0.0004 (12)
C10	0.0106 (18)	0.0124 (16)	0.0090 (15)	0.0034 (13)	0.0008 (13)	0.0010 (12)
O10	0.045 (2)	0.046 (2)	0.0290 (17)	−0.0029 (16)	0.0128 (15)	−0.0107 (14)

### Geometric parameters (Å, °)

**Table:**

Gd1—O6^i^	2.337 (3)	C2—C4	1.396 (5)
Gd1—O7^ii^	2.376 (2)	O3—K1^ii^	3.306 (3)
Gd1—O8	2.433 (2)	C3—C4^vii^	1.394 (4)
Gd1—O3	2.441 (2)	C3—H3	0.9300
Gd1—O1	2.447 (3)	O4—C5	1.253 (4)
Gd1—O5^iii^	2.451 (2)	O4—Gd1^iv^	2.503 (2)
Gd1—O4^iii^	2.503 (2)	O4—K1^iii^	2.875 (3)
Gd1—O9	2.520 (3)	C4—C3^vii^	1.394 (4)
Gd1—O2	2.604 (2)	C4—C5	1.502 (5)
Gd1—C6	2.825 (3)	O5—C5	1.266 (4)
Gd1—C5^iii^	2.831 (3)	O5—Gd1^iv^	2.451 (2)
Gd1—C1	2.882 (3)	C5—Gd1^iv^	2.831 (3)
K1—O2	2.714 (3)	O6—C10	1.259 (4)
K1—O6	2.783 (3)	O6—Gd1^v^	2.337 (3)
K1—O8	2.795 (3)	C6—O9	1.249 (4)
K1—O10	2.842 (3)	C6—O8	1.261 (4)
K1—O1^iv^	2.860 (3)	C6—C7	1.504 (4)
K1—O4^iv^	2.875 (3)	O7—C10	1.253 (4)
K1—O7^i^	2.891 (3)	O7—Gd1^vi^	2.376 (2)
K1—O9^v^	2.893 (3)	O7—K1^v^	2.891 (3)
K1—C10	3.230 (3)	C7—C8	1.392 (4)
K1—O3^vi^	3.306 (3)	C7—C9	1.402 (5)
K1—Gd1^v^	3.9912 (10)	C8—C9^viii^	1.396 (4)
O1—K1^iii^	2.860 (3)	C8—H8	0.9300
O1—H1A	0.8501	O9—K1^i^	2.893 (3)
O1—H1B	0.8500	C9—C8^viii^	1.396 (4)
C1—O2	1.250 (4)	C9—C10	1.503 (4)
C1—O3	1.263 (4)	O10—H10A	0.8671
C1—C2	1.506 (4)	O10—H10B	0.8656
C2—C3	1.388 (5)		
			
O6^i^—Gd1—O7^ii^	72.72 (8)	O6—K1—O3^vi^	105.99 (7)
O6^i^—Gd1—O8	94.93 (9)	O8—K1—O3^vi^	129.52 (7)
O7^ii^—Gd1—O8	123.50 (8)	O10—K1—O3^vi^	79.96 (8)
O6^i^—Gd1—O3	78.90 (9)	O1^iv^—K1—O3^vi^	52.77 (7)
O7^ii^—Gd1—O3	105.04 (9)	O4^iv^—K1—O3^vi^	52.76 (7)
O8—Gd1—O3	126.89 (8)	O7^i^—K1—O3^vi^	140.46 (8)
O6^i^—Gd1—O1	140.86 (9)	O9^v^—K1—O3^vi^	81.54 (7)
O7^ii^—Gd1—O1	145.14 (9)	C10—K1—O3^vi^	88.80 (7)
O8—Gd1—O1	72.49 (9)	O2—K1—Gd1^v^	109.12 (6)
O3—Gd1—O1	79.97 (9)	O6—K1—Gd1^v^	34.92 (5)
O6^i^—Gd1—O5^iii^	149.95 (9)	O8—K1—Gd1^v^	88.21 (6)
O7^ii^—Gd1—O5^iii^	78.16 (8)	O10—K1—Gd1^v^	105.38 (8)
O8—Gd1—O5^iii^	94.98 (8)	O1^iv^—K1—Gd1^v^	155.64 (6)
O3—Gd1—O5^iii^	116.41 (8)	O4^iv^—K1—Gd1^v^	113.43 (6)
O1—Gd1—O5^iii^	69.16 (9)	O7^i^—K1—Gd1^v^	36.12 (5)
O6^i^—Gd1—O4^iii^	121.37 (8)	O9^v^—K1—Gd1^v^	38.99 (5)
O7^ii^—Gd1—O4^iii^	71.02 (8)	C10—K1—Gd1^v^	54.57 (6)
O8—Gd1—O4^iii^	143.63 (9)	O3^vi^—K1—Gd1^v^	109.62 (5)
O3—Gd1—O4^iii^	68.24 (8)	O2—K1—Gd1	37.34 (5)
O1—Gd1—O4^iii^	79.53 (9)	O6—K1—Gd1	82.38 (5)
O5^iii^—Gd1—O4^iii^	52.70 (8)	O8—K1—Gd1	34.09 (5)
O6^i^—Gd1—O9	81.44 (9)	O10—K1—Gd1	108.80 (7)
O7^ii^—Gd1—O9	71.09 (8)	O1^iv^—K1—Gd1	111.26 (6)
O8—Gd1—O9	52.45 (8)	O4^iv^—K1—Gd1	106.89 (5)
O3—Gd1—O9	160.19 (9)	O7^i^—K1—Gd1	63.78 (5)
O1—Gd1—O9	114.70 (9)	O9^v^—K1—Gd1	122.78 (6)
O5^iii^—Gd1—O9	82.34 (9)	C10—K1—Gd1	92.00 (6)
O4^iii^—Gd1—O9	125.60 (8)	O3^vi^—K1—Gd1	155.35 (6)
O6^i^—Gd1—O2	70.03 (8)	Gd1^v^—K1—Gd1	90.638 (18)
O7^ii^—Gd1—O2	139.01 (8)	Gd1—O1—K1^iii^	135.72 (11)
O8—Gd1—O2	76.60 (8)	Gd1—O1—H1A	104.3
O3—Gd1—O2	51.49 (8)	K1^iii^—O1—H1A	61.0
O1—Gd1—O2	71.01 (9)	Gd1—O1—H1B	103.4
O5^iii^—Gd1—O2	139.97 (9)	K1^iii^—O1—H1B	120.7
O4^iii^—Gd1—O2	115.76 (8)	H1A—O1—H1B	107.7
O9—Gd1—O2	118.52 (8)	O2—C1—O3	121.8 (3)
O6^i^—Gd1—C6	90.28 (9)	O2—C1—C2	120.9 (3)
O7^ii^—Gd1—C6	97.28 (10)	O3—C1—C2	117.1 (3)
O8—Gd1—C6	26.43 (9)	O2—C1—Gd1	64.62 (18)
O3—Gd1—C6	150.81 (9)	O3—C1—Gd1	57.19 (17)
O1—Gd1—C6	92.43 (10)	C2—C1—Gd1	171.3 (2)
O5^iii^—Gd1—C6	86.17 (9)	C1—O2—Gd1	89.7 (2)
O4^iii^—Gd1—C6	138.40 (8)	C1—O2—K1	165.2 (2)
O9—Gd1—C6	26.23 (9)	Gd1—O2—K1	103.46 (8)
O2—Gd1—C6	99.35 (8)	C3—C2—C4	118.7 (3)
O6^i^—Gd1—C5^iii^	141.71 (9)	C3—C2—C1	116.3 (3)
O7^ii^—Gd1—C5^iii^	74.19 (9)	C4—C2—C1	125.0 (3)
O8—Gd1—C5^iii^	119.44 (10)	C1—O3—Gd1	97.0 (2)
O3—Gd1—C5^iii^	91.69 (9)	C1—O3—K1^ii^	155.9 (2)
O1—Gd1—C5^iii^	71.17 (9)	Gd1—O3—K1^ii^	93.34 (8)
O5^iii^—Gd1—C5^iii^	26.51 (9)	C2—C3—C4^vii^	121.8 (3)
O4^iii^—Gd1—C5^iii^	26.26 (9)	C2—C3—H3	119.1
O9—Gd1—C5^iii^	105.40 (9)	C4^vii^—C3—H3	119.1
O2—Gd1—C5^iii^	130.73 (9)	C5—O4—Gd1^iv^	91.6 (2)
C6—Gd1—C5^iii^	112.63 (10)	C5—O4—K1^iii^	148.9 (2)
O6^i^—Gd1—C1	72.32 (10)	Gd1^iv^—O4—K1^iii^	103.25 (9)
O7^ii^—Gd1—C1	123.98 (9)	C3^vii^—C4—C2	119.5 (3)
O8—Gd1—C1	101.85 (9)	C3^vii^—C4—C5	117.5 (3)
O3—Gd1—C1	25.79 (9)	C2—C4—C5	123.0 (3)
O1—Gd1—C1	74.32 (10)	C5—O5—Gd1^iv^	93.7 (2)
O5^iii^—Gd1—C1	132.62 (10)	O4—C5—O5	121.6 (3)
O4^iii^—Gd1—C1	92.23 (9)	O4—C5—C4	119.8 (3)
O9—Gd1—C1	141.67 (9)	O5—C5—C4	118.6 (3)
O2—Gd1—C1	25.71 (8)	O4—C5—Gd1^iv^	62.10 (18)
C6—Gd1—C1	125.03 (10)	O5—C5—Gd1^iv^	59.77 (18)
C5^iii^—Gd1—C1	112.55 (10)	C4—C5—Gd1^iv^	174.2 (2)
O2—K1—O6	116.27 (8)	C10—O6—Gd1^v^	138.0 (2)
O2—K1—O8	69.09 (7)	C10—O6—K1	99.1 (2)
O6—K1—O8	63.05 (8)	Gd1^v^—O6—K1	102.10 (9)
O2—K1—O10	72.67 (9)	O9—C6—O8	121.5 (3)
O6—K1—O10	140.08 (10)	O9—C6—C7	119.1 (3)
O8—K1—O10	141.74 (9)	O8—C6—C7	119.1 (3)
O2—K1—O1^iv^	84.58 (8)	O9—C6—Gd1	63.11 (18)
O6—K1—O1^iv^	153.86 (8)	O8—C6—Gd1	59.14 (17)
O8—K1—O1^iv^	115.86 (9)	C7—C6—Gd1	166.0 (2)
O10—K1—O1^iv^	58.46 (9)	C10—O7—Gd1^vi^	147.2 (2)
O2—K1—O4^iv^	123.48 (8)	C10—O7—K1^v^	114.5 (2)
O6—K1—O4^iv^	83.45 (7)	Gd1^vi^—O7—K1^v^	98.05 (8)
O8—K1—O4^iv^	76.77 (7)	C8—C7—C9	119.4 (3)
O10—K1—O4^iv^	125.96 (9)	C8—C7—C6	117.3 (3)
O1^iv^—K1—O4^iv^	71.42 (8)	C9—C7—C6	123.2 (3)
O2—K1—O7^i^	73.25 (8)	C6—O8—Gd1	94.4 (2)
O6—K1—O7^i^	58.96 (7)	C6—O8—K1	127.7 (2)
O8—K1—O7^i^	79.25 (8)	Gd1—O8—K1	105.81 (9)
O10—K1—O7^i^	90.92 (9)	C7—C8—C9^viii^	120.9 (3)
O1^iv^—K1—O7^i^	146.80 (8)	C7—C8—H8	119.5
O4^iv^—K1—O7^i^	141.64 (7)	C9^viii^—C8—H8	119.5
O2—K1—O9^v^	120.23 (8)	C6—O9—Gd1	90.7 (2)
O6—K1—O9^v^	67.91 (7)	C6—O9—K1^i^	136.5 (2)
O8—K1—O9^v^	127.17 (8)	Gd1—O9—K1^i^	94.77 (8)
O10—K1—O9^v^	74.28 (9)	C8^viii^—C9—C7	119.7 (3)
O1^iv^—K1—O9^v^	116.76 (8)	C8^viii^—C9—C10	118.6 (3)
O4^iv^—K1—O9^v^	116.29 (8)	C7—C9—C10	121.4 (3)
O7^i^—K1—O9^v^	59.01 (7)	O7—C10—O6	123.9 (3)
O2—K1—C10	129.24 (8)	O7—C10—C9	119.4 (3)
O6—K1—C10	22.64 (8)	O6—C10—C9	116.7 (3)
O8—K1—C10	63.26 (8)	O7—C10—K1	91.9 (2)
O10—K1—C10	152.09 (10)	O6—C10—K1	58.30 (18)
O1^iv^—K1—C10	131.40 (9)	C9—C10—K1	121.7 (2)
O4^iv^—K1—C10	60.96 (8)	K1—O10—H10A	138.3
O7^i^—K1—C10	81.59 (8)	K1—O10—H10B	102.5
O9^v^—K1—C10	78.87 (8)	H10A—O10—H10B	106.3
O2—K1—O3^vi^	137.10 (8)		

Symmetry codes: (i) −*x*, *y*−1/2, −*z*+3/2; (ii) *x*, *y*−1, *z*; (iii) −*x*+1, *y*−1/2, −*z*+3/2; (iv) −*x*+1, *y*+1/2, −*z*+3/2; (v) −*x*, *y*+1/2, −*z*+3/2; (vi) *x*, *y*+1, *z*; (vii) −*x*+1, −*y*+1, −*z*+2; (viii) −*x*, −*y*+2, −*z*+1.
